# DNA methylation biomarkers for nasopharyngeal carcinoma

**DOI:** 10.1371/journal.pone.0230524

**Published:** 2020-04-09

**Authors:** Baoai Han, Xiuping Yang, Po Zhang, Ya Zhang, Yaqin Tu, Zuhong He, Yongqin Li, Jie Yuan, Yaodong Dong, Davood K. Hosseini, Tao Zhou, Haiying Sun

**Affiliations:** 1 Public Laboratory, Key Laboratory of Breast Cancer Prevention and Therapy, Ministry of Education, Tianjin Medical University Cancer Institute and Hospital, National Clinical Research Center for Cancer, Tianjin Medical University, Tianjin, China; 2 Department of Otorhinolaryngology, Head and Neck Surgery, Zhongnan Hospital of Wuhan University, Wuhan, China; 3 Department of Neurosurgery Tongji Hospital, Tongji Medical College, Huazhong University of Science and Technology, Wuhan, China; 4 Department of Otorhinolaryngology, Union Hospital, Tongji Medical College, Huazhong University of Science and Technology, Wuhan, China; 5 Department of Otolaryngology-Head and Neck Surgery, Stanford University School of Medicine, Stanford, California, United States of America; 6 Department of Medicine, Stanford University School of Medicine, Stanford, California, United States of America; University of Calgary, CANADA

## Abstract

**Background:**

Aberrant methylation of DNA plays an important role in the pathogenesis of nasopharyngeal carcinoma (NPC). In the current study, we aimed to integrate three cohorts profile datasets to identify abnormally methylated-differentially expressed genes and pathways associated with NPC.

**Methods:**

Data of gene expression microarrays (GSE53819, GSE412452) and gene methylation microarrays (GSE52068) obtained from the GEO database. Aberrantly methylated differentially expressed genes (DEGs) were obtained by GEO2R. The David database was utilized to perform enrichment and functional analysis regarding selected genes. To create a protein-protein interaction (PPI), STRING and Cytoscape software were utilized. The MCODE was used for module analysis of the PPI network.

**Results:**

In total, 181 hypomethylation-high expression genes were identified, which were enriched in the biological mechanisms involved in the differentiation of endodermal cell, mitotic nuclear division, mitotic cell cycle process, chromosome segregation and cell cycle phase transition, etc. Pathway enrichment showed ECM-receptor interaction, PI3K-Akt signaling pathway, Focal adhesion, Protein digestion and absorption and Amoebiasis, etc. The top 3 hub genes of PPI network were FANCI, POSTN, and IFIH1. Additionally, 210 hypermethylation-low expression genes were identified, and our data revealed enrichment in biological processes including axoneme assembly, micro tubular formation, assembly of axonemal dynein complex, cilium movement and cilium organization, etc. Pathway analysis indicated enrichment in B cell receptor signaling pathway, Hematopoietic cell lineage, Leukocyte transendothelial migration, Complement and coagulation cascades and Fc gamma R-mediated phagocytosis, etc. The ZMYND10, PACRG and POU2AF1 were identified as the top three hub genes of PPI network. After validation in TCGA and GEPIA database, most hub genes remained significant. Patients with high expression of POSTN found to have shorter overall survival, while in patients with high expression of ZMYND10 and POU2AF1 longer overall survival was identified.

**Conclusions:**

The data revealed novel aberrantly methylated-differentially expressed genes and pathways in NPC by bioinformatics analysis, potentially providing novel insights for the molecular mechanisms governing NPC progression. Hub genes including FANCI, POSTN, IFIH1, ZMYND10, PACRG and POU2AF1 might serve as novel biomarkers for precision diagnosis and providing medical treatment for patient with NPC.

## Background

Nasopharyngeal carcinoma (NPC) is a kind of cancer arising from the nasopharynx epithelium cells and it is one of the most common malignant tumors in the head and neck [1]. It is common in southern China, with poorly differentiated or undifferentiated type and high degree of malignancy. In contrast, most of the nasopharyngeal carcinomas in western countries are highly differentiated and the degree of malignancy is relatively low [2]. Although NPC is sensitive to radiotherapy and adjuvant therapy and has a 5-year survival rate of about 70% [3], recurrence and metastasis are still difficult problems in current treatment. At present, the etiology of nasopharyngeal carcinoma includes three aspects: environmental factors, genetic susceptibility and Epstein-Barr virus (EBV) infection [4,5,6]. Increased expression of oncogenes and decreased expression of tumor suppressor genes are the key factors for tumorigenesis. However the mechanism of the progression of NPC is not fully studied and described. Thus, further investigation is beneficial, especially for identification of potential biomarkers to improve survival in patients whom their NPC are in early-stages.

The term epigenetics refers to alterations in the expression of hereditary genes unrelated to DNA sequence changes [7,8]. DNA methylation is the most common epigenetic change, and methylation changes in promoter or first exon CpG island can lead to inactivation of gene expression [9]. DNA methylation is closely related to gene expression regulation [10], embryonic development [11], X chromosome inactivation [12], genomic stability [13] and genomic imprinting [14]. DNA methylation is more common in patients with EB virus-associated NPC [15]. Abnormal DNA methylation affects the function of key genes, especially tumour suppressor genes, and thus participates in various processes of nasopharyngeal carcinoma development. Although many studies detected certain genes with abnormal DNA hypermethylation or hypomethylation in nasopharyngeal carcinoma, it is still difficult to determine the comprehensive profile and pathways of the interaction network.

Comprehensive analysis of multiple datasets provides the capabilities to properly identify and assess the pathways and genes that mediate the biological processes associated with nasopharyngeal carcinoma. To this end, we used microarray datasets of gene expression (GSE53819, GSE12452) and gene methylation (GSE52068) to assess nasopharyngeal carcinoma genes and epigenetic features in order to identify abnormally methylated genes and pathways, and thus differentially expressed. Utilizing of a protein-protein interaction network we were also capable to identify key so-called "hub" genes that were key to these signalling events. Furthermore, we validated the results using the Cancer Genome Atlas (TCGA) data to identify differentially methylated genes (DMGs) involved in the pathogenesis of NPC. Finally, GEPIA database was utilized to perform survival analysis. In summery we believe that it is feasible to identify novel differentially methylated genes associated with NPC with the explained method, providing key insights into the NPC at the molecular level that governed the NPC development and progressionto full blown stages.

## Methods

### Microarray data

In our study, the gene expression profiling datasets GSE53819 and GSE12452, and gene methylation profiling datasets GSE52068 were obtained from the gene expression omnibus (GEO, https://www.ncbi.nlm.nih.gov/geo/) [16,17]. Totally 18NPC and 18 normal specimens were obtained in GSE53819 while 31NPC and 10 normal samples were obtained in GSE12452. Both expression microarrays used the platform GPL570 (Affymetrix Human Genome U133 plus 2.0 Array, Thermo Fisher scientific, USA). For the gene methylation profiling microarray, GSE52068 included a total of 24 primary NPC tumour samples and 24 normal nasopharyngeal epithelial tissue samples. The platform of this methylation microarray was GPL13534 (Illumina Human Methylation Bead Chip).

### Data acquisition and processing

We utilized GEO2R online software to analysis the raw submitter-supplied data of microarrays and identify differential methylated genes (DMGs) and differentially expressed genes (DEGs). GEO2R is an interactive web instrument allowing comparison of different groups of samples in a GEO series in order to examine differentially expressed genes according to experimental conditions. | log FC | ≥ 1 and P < 0.05 were used as the cut-off standards to obtain DEGs and |t| > 2 and P < 0.05 were used as the cut-off standards to find DMGs. Eventually, a Venn diagram was used to identify hypermethylated lowly-expressed genes and hypomethylated highly expressed genes.

### Functional and pathway enrichment analysis

Gene ontology (GO) analysis including the molecular function, biological process and cellular component and Kyoto Encyclopaedia of Genes and Genomes (KEGG) pathway enrichment analysis were conducted on selected genes with hypomethylation-high expression and hypermethylation-low expression by DAVID (DAVID, https://david.ncifcrf.gov/). P < 0.05 was regarded as statistical significance.

### Generation and analysis of a protein–protein interaction (PPI) network

The functional PPI analysis is essential to interpret the molecular mechanisms of key cellular activities in carcinogenesis. In our study, we used STRING (https://string-db.org/) database to construct PPI network of hypomethylation/high-expression genes and hypermethylation/low-expression genes, respectively. Interaction score of 0.4 was regarded as the cut-off criterion and the PPI was visualized. Then, the Molecular Complex Detection (MCODE) in Cytoscape software was conducted to screen modules within PPI network with MCODE score >3 and number of nodes >4. The top 3 Hub genes were chosen by CytoHubba app in Cytoscape software. To determine the expression pattern of six hub genes in NPC, the datasets was used in the Oncomine (https://www.oncomine.org). Oncomine is an online database consisting of previously published and open-access microarray data. The analysis enables multiple comparisons of gene expression between different researches; the importance of the gene expression across the available researches was also taken into account [18,19]. The consequences were filtered by selecting nasopharyngeal carcinoma vs. normal tissue.

### Hub gene validation

The Cancer Genome Atlas (TCGA) database, collaboration between the National Cancer Institute and National Human Genome Research Institute, has provided comprehensive, multi-dimensional maps of the key genomic changes for the variety of malignancies. MEXPRESS (http://mexpress.be/) is a validated dataset designed for the easy visualization of TCGA expression, DNA methylation and clinical data, as well as the relationships between them [20]. We utilized the MEXPRESS to validate hypermethylation/low-expression hub genes and hypomethylation/high-expression hub genes in TCGA database and confirm our findings. The probability of survival and significance was calculated using the GEPIA database. GEPIA is a newly created online interactive web server which enables users to explore the RNA sequencing expression information of tumors/normal tissues or samples from the Genotype Tissue Expression (GTEx) projects and The Cancer Genome Atlas (TCGA), based on a criterion processing pipeline. GEPIA offer customizable functions such as profiling regarding to pathological stages, cancer types, differential expression analysis, survival analysis, correlation analysis and similar gene detection.

## Results

### Identification of abnormally methylated and differentially expressed genes in NPC

**Fig 1:**
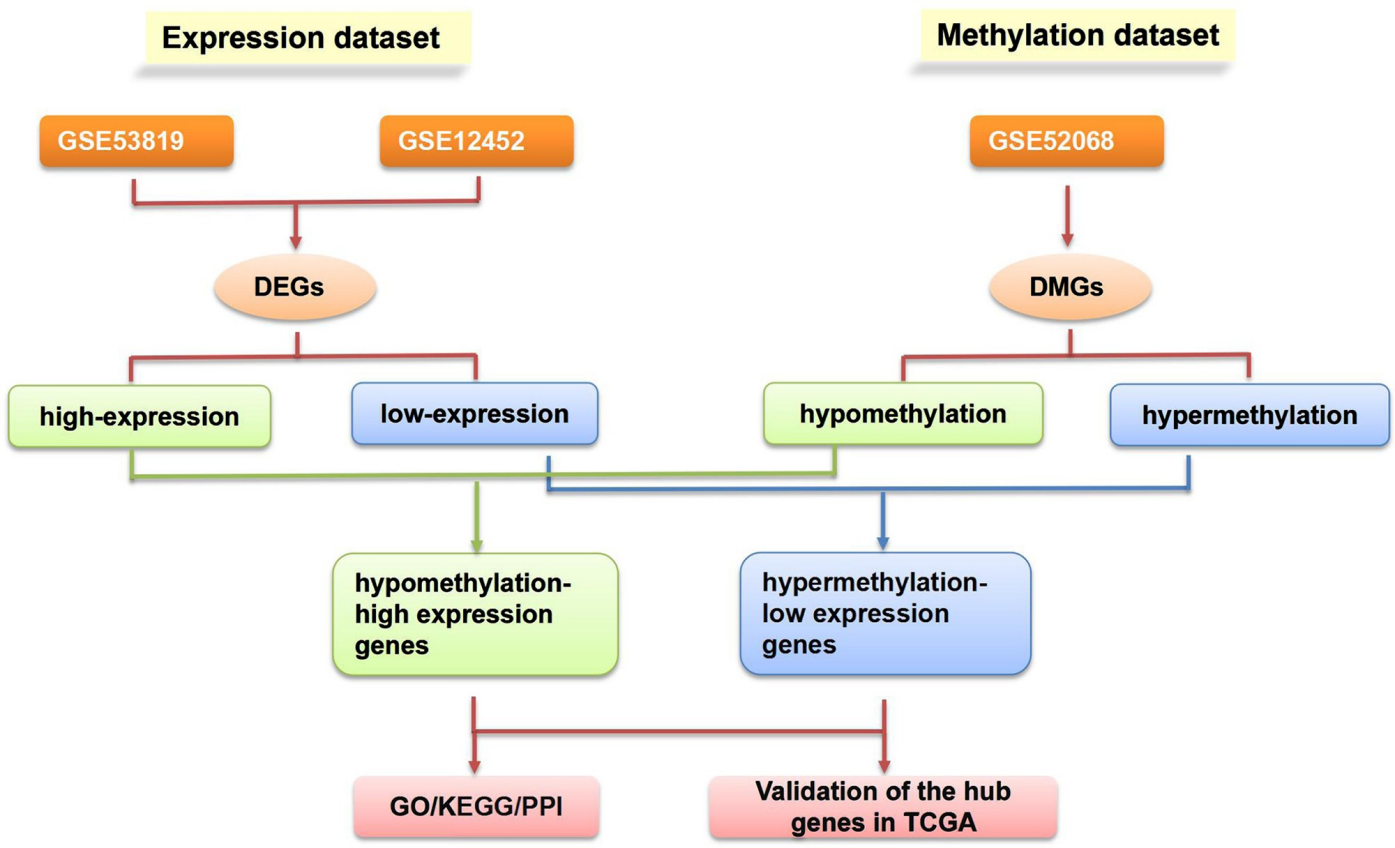
The flowchart of our study. DMG: differentially methylated gene; DEG: differentially expressed gene; GO: gene ontology; KEGG: Kyoto Encyclopedia of Genes and Genomes; PPI: protein-protein interaction; TCGA: the Cancer Genome Atlas.

**Fig 2:**
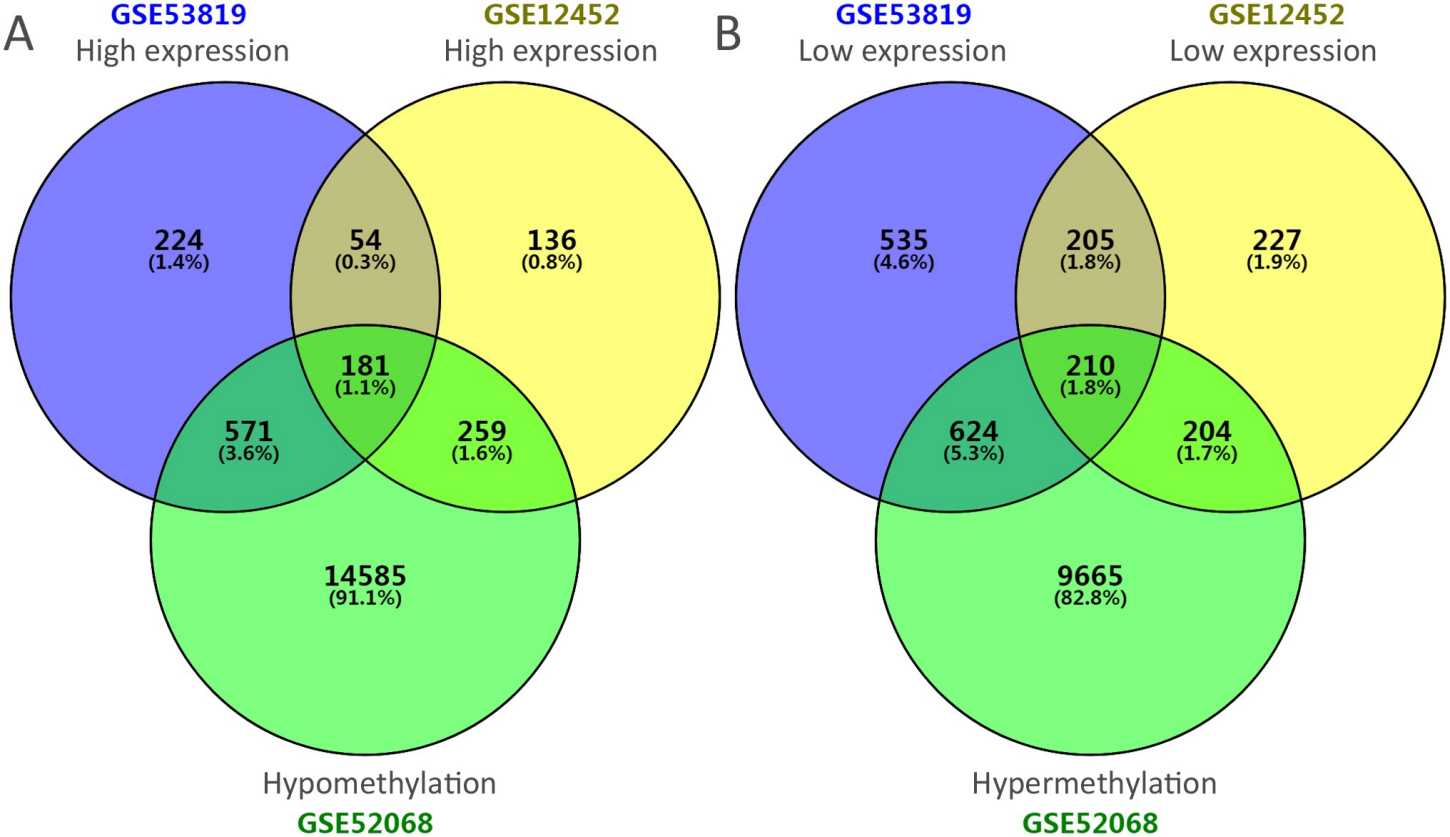
Funrich software utilized for identification of aberrantly methylated and differentially expressed genes. We used different color to demonstrate deferent datasets. A: Hypomethylation and high expression genes; B: hypermethylation and low expression genes.

**Fig 3:**
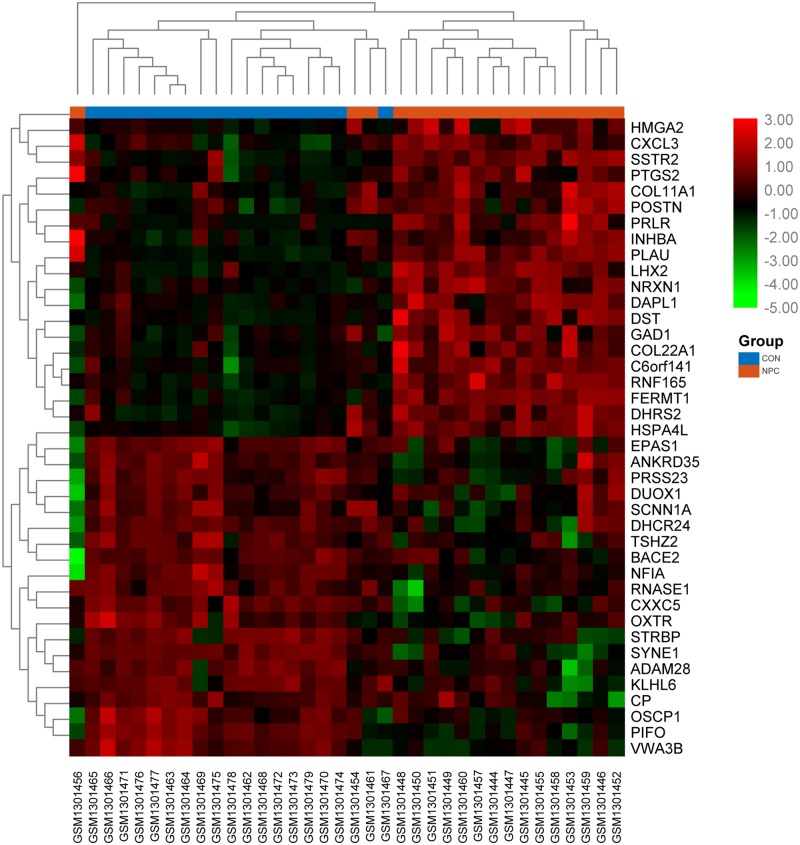
The heat map of top 20 hypomethylation/high-expression genes and top 20 hypermethylation/low-expression genes in GSE53819.

The flowchart of our study is demonstrated in Fig 1. Assessing the DEGs of the gene expression microarray, we identified 235 overlapping upregulated genes (1030 in GSE53819 and 630 in GSE12452) and 415 overlapping downregulated genes (1574 in GSE53819 and 846 in GSE12452). Assessing DMGs of the gene methylation microarray, we found 10703 hypermethylation genes and 15596 hypomethylation genes. In addition, a total of 181 hypomethylation/high-expression genes were obtained by overlapping 15596 hypomethylation genes and 235 upregulated genes; As Venn diagram show, 210 hypermethylation/low- expression genes were obtained by overlapping 10703 hypermethylation genes and 415 downregulated genes (Fig 2). The heat map of top 20 hypomethylation/high- expression genes and top 20 hypermethylation/low- expression genes in GSE53819 are demonstrated in Fig 3.

### Gene ontology and pathway functional enrichment analysis

**Fig 4:**
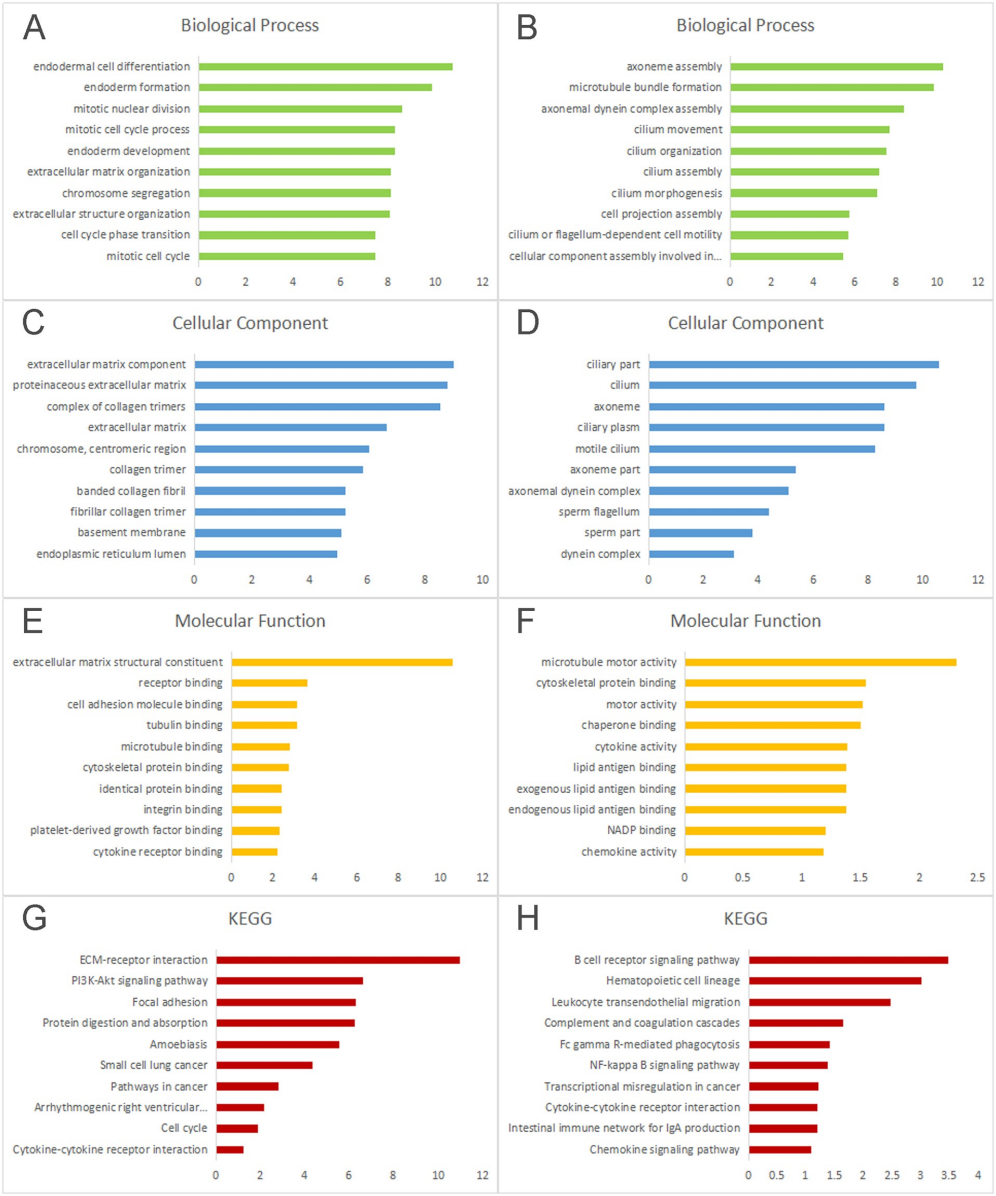
The gene ontology annotation and pathway enrichment analysis and demonstration of aberrantly methylated and differentially expressed genes. A: Biological process, C: cellular component, E: molecular function, and G: KEGG of hypomethylation/high-expression genes. B: Biological process, D: cellular component, F: molecular function, and H: KEGG of hypermethylation/low-expression genes. The high enrichment score means that the genes were found more frequently in the particular ontology. KEGG, Kyoto Encyclopedia of Genes and Genomes.

The GO annotation and pathway enrichment analyses of selected aberrantly methylated and differentially expressed genes were implemented using the online tool DAVID. Genes that were hypomethylated and highly expressed were enriched for endodermal cell differentiation, mitotic nuclear division, mitotic cell cycle process, chromosome segregation and cell cycle phase transition (Fig 4A), while the hypermethylated low-expression genes were primarily linked to formation of axoneme, formation of microtubule bundle, assembly of axonemal dynein complex, cilium movement and cilium organization (Fig 4B). Analysis of the cell component enrichment revealed that hypomethylated highly-expressed genes were correlated with extracellular matrix component, proteinaceous extracellular matrix, complex of collagen trimers, extracellular matrix, chromosome and centromeric region (Fig 4C), whereas hypermethylation/low-expression genes were predominant at ciliary part, cilium, axoneme, ciliary plasm and motile cilium (Fig 4D). As for molecular function, hypomethylation/high-expression genes were enriched mainly in extracellular matrix structural constituent, receptor binding, cell adhesion molecule binding, tubulin binding and microtubule binding (Fig 4E), while hypermethylation/low-expression genes were mostly enriched in microtubule motor activity, cytoskeletal protein binding, motor activity, chaperone binding and cytokine activity (Fig 4F). The pathway analysis revealed that hypomethylated highly-expressed genes were linked to ECM-receptor interaction, PI3K-Akt signaling pathway, Focal adhesion, Protein digestion and absorption and Amoebiasis (Fig 4G), while hypermethylation/low-expression genes significantly enriched in B cell receptor signaling pathway, Hematopoietic cell lineage, Leukocyte transendothelial migration, Complement and coagulation cascades and Fc gamma R-mediated phagocytosis (Fig 4H). These screened pathways demonstrated that DEGs and DMGs have a crucial function in the tumor microenvironment, etiology and pathogenesis in NPC.

### Construction and analysis of PPI networks

**Fig 5:**
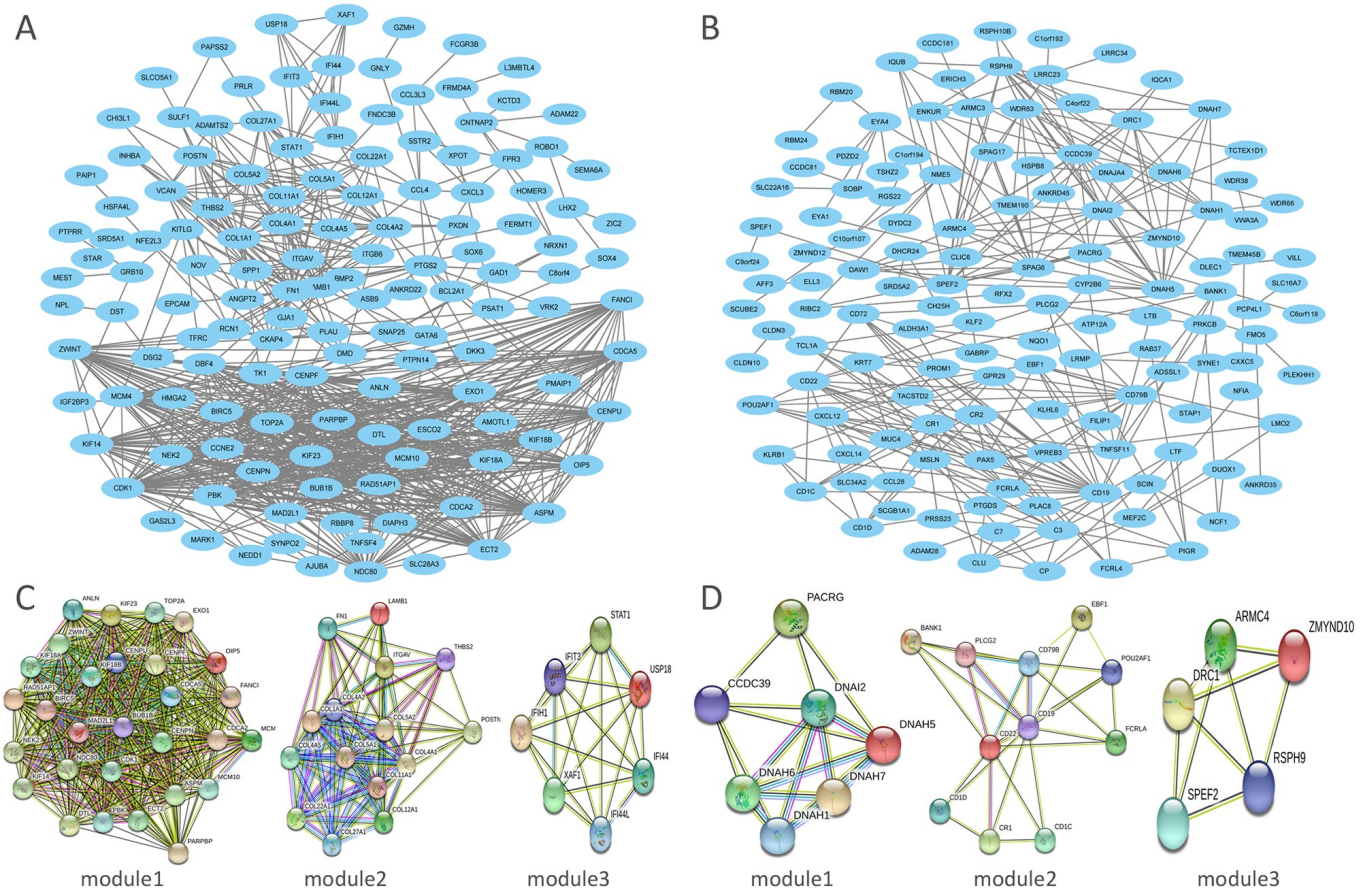
PPI network and top 3 modules of aberrantly methylated and differentially expressed genes. A: PPI network of hypomethylation/high-expression genes and B: PPI network of hypermethylation/low-expression genes. C: Top 3 modules of hypomethylation/ high-expression genes and D: Top 3 modules of hypomethylation/low-expression genes. PPI, protein-protein interaction.

**Fig 6:**
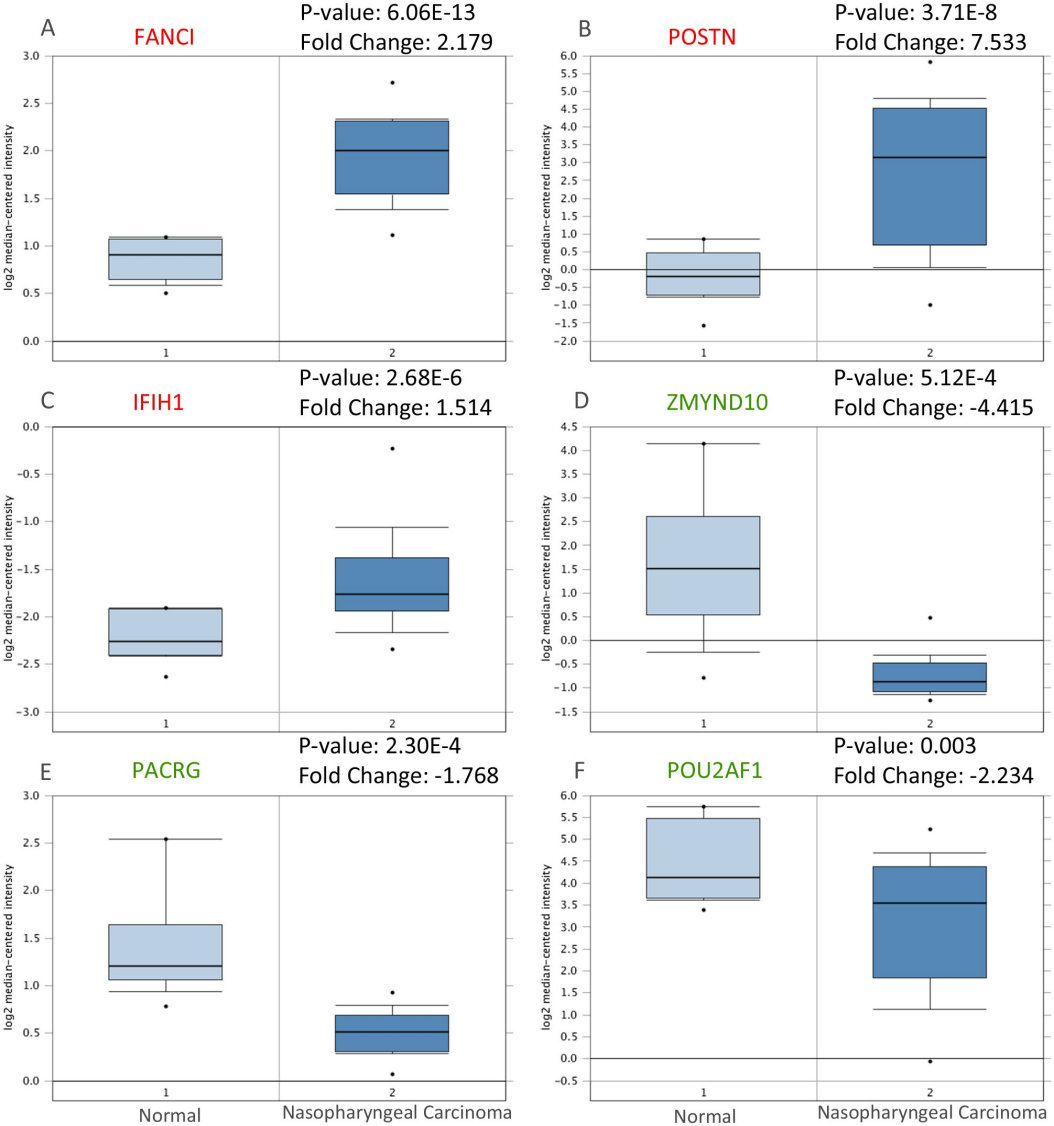
Validation of the expression of hub genes in Oncomine database. The expression level of A: FANCI, B: POSTN, C: IFIH1, D: ZMYND, E: PACRG, and F: POU2AF1 were detected in Oncomine database. Red: Hypomethylation/high-expression genes; Green: Hypermethylation/low-expression genes.

The STRING database was applied for PPI network construction, with MCODE applied for module analysis. Hub genes were chose using the cyto Hubba Cytoscape software. The PPI network for genes that were hypomethylated and highly expressed is demonstrated in Fig 5A, with corresponding modules shown in Fig 5C. The most significantly enriched functional modules were those linked to Cell cycle, Protein digestion and absorption, ECM-receptor interaction, Focal adhesion, Hepatitis B, Measles and Influenza A (Table 1). Top three hub genes were FANCI, POSTN and IFIH1. The PPI network for genes that were hypermethylated and expressed at low levels is demonstrated in Fig 5B, with corresponding modules demonstrated in Fig 5D. Significant modules showed functions including Huntington's disease, Hematopoietic cell lineage and B cell receptor signal pathway (Table 1). Top three hub genes were ZMYND10, PACRG and POU2AF1. Furthermore, we use Oncomine database to confirm the expression of hub genes in NPC (Fig 6). The data is consistent with our outcomes.

**Table 1:** Module analysis of the protein-protein interaction network.

Category	Module	Pathway description	FDR	Nodes	Genes
**Hypomethylation and high expression**					
	1	Cell cycle	5.30E-04	4	BUB1B,CDK1,MAD2L1,MCM4
	2	Protein digeston and absorption	4.92E-19	10	COL11A1,COL12A1,COL1A1,COL22A1, COL27A1,COL4A,COL4A2,COL4A5, COL5A1,COL5A2
		ECM-receptor interaction	2.00E-12	7	COL1A1,COL4A1,COL4A2,COL4A5, ITGAV,LAMB1,THBS2
		Focal adhesion	5.28E-10	7	COL1A1,COL4A1,COL4A2,COL4A5, ITGAV,LAMB1,THBS2
		Human papillomavirus infection	1.01E-08	7	COL1A1,COL4A1,COL4A2,COL4A5, ITGAV,LAMB1,THBS2
		PI3K-Akt signaling pathway	1.52E-08	7	COL1A1,COL4A1,COL4A2,COL4A5, ITGAV,LAMB1,THBS2
		Amoebiasis	2.85E-08	5	COL1A1,COL4A1,COL4A2,COL4A5, LAMB1
		Small cell lung cancer	2.85E-08	5	COL4A1,COL4A2,COL4A5,ITGAV, LAMB1
		AGE-RAGE signaling pathway in diabetic complications	2.49E-06	4	COL1A1,COL4A1,COL4A2,COL4A5
		Relaxin signaling pathway	6.60E-06	4	COL1A1,COL4A1,COL4A2,COL4A5
		Pathways in cancer	6.87E-05	5	COL4A1,COL4A2,COL4A5,ITGAV,LAMB1
		Phagosome	1.10E-02	2	ITGAV,THBS2
	3	Hepatitis B	2.42E-02	2	IFIH1,STAT1
		Measles	2.42E-02	2	IFIH1,STAT1
		Influenza A	2.42E-02	2	IFIH1,STAT1
		Herpes simplex infection	2.42E-02	2	IFIH1,STAT1
**Hypermethylation and low expression**					
	1	Huntington's disease	2.08E-09	5	DNAH1,DNAH5,DNAH6,DNAH7,DNAI2
	2	Hematopoietic cell lineage	5.93E-08	5	CD19,CD1C,CD1D,CD22,CR1
		B cell receptor signaling pathway	1.41E-06	4	CD19,CD22,CD79B,PLCG2

### Validation of the hub genes

**Fig 7:**
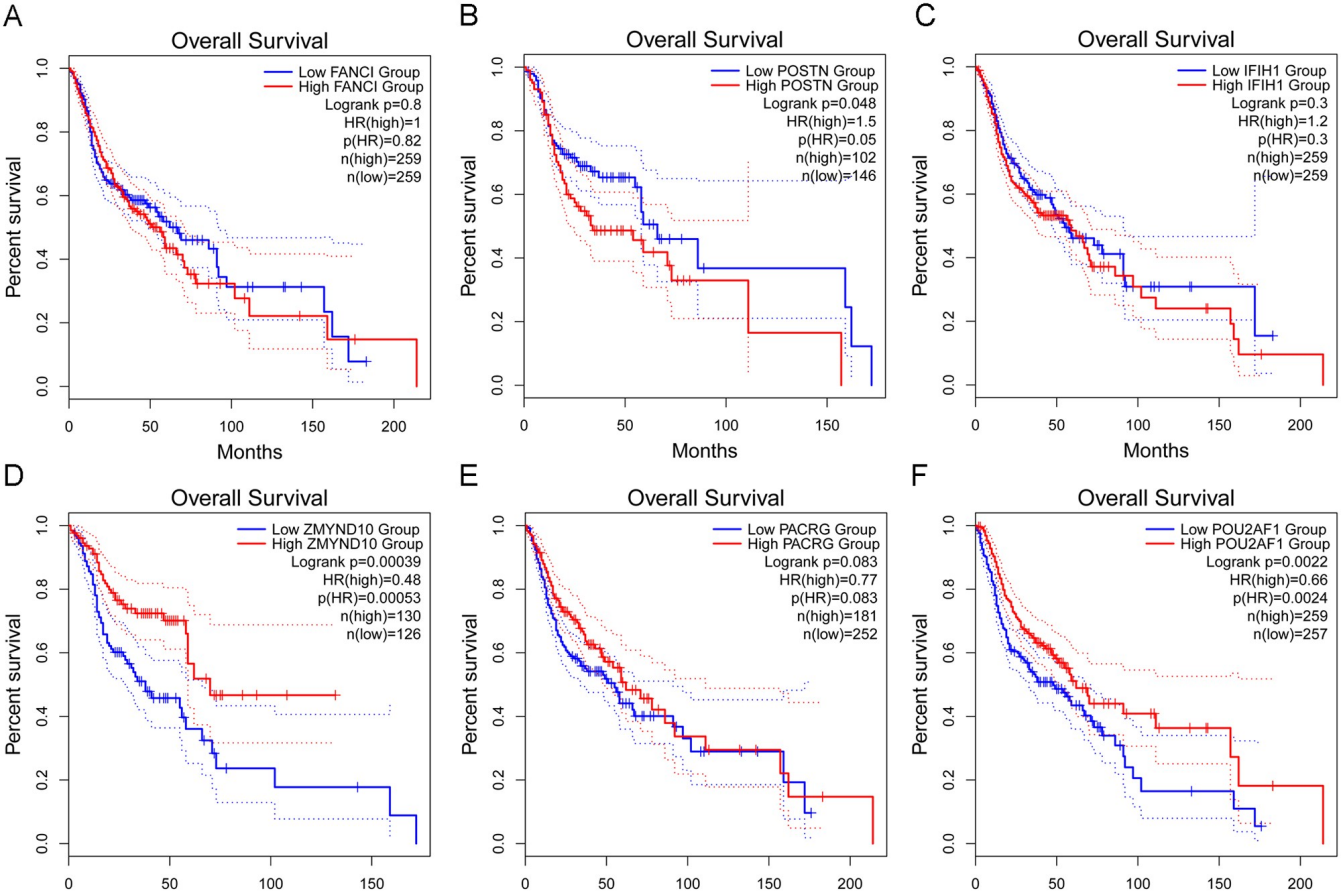
Prognostic value of six hub genes in NPC. Prognostic value of A: FANCI, B: POSTN, C: IFIH1, D: ZMYND10, E: PACRG, and F: POU2AF1 were detected in GEPIA database. The survival curve comparing the patients with high (red) and low (blue) expression in NPC.

Hypermethylation-low expression hub genes and hypomethylation-high expression hub genes were then validated in another database TCGA to confirm the outcomes. The outcomes were summed up in Table 2. For most of the hub genes, the expression and methylation status were still significantly varying among given groups and same with our outcomes, which indicate the reliability and stability of the findings. Then, we tried to analysis the relationship between hub genes and the survival in NPC. The prognostic value of six hub genes was assessed by GEPIA database. Patients with high expression of POSTN were associated with shorter overall survival, while patients with high expression of ZMYND10 and POU2AF1 were associated with longer overall survival (Fig 7). Although P-value is not significant, it has survival significance from the perspective of picture trend that patients with high expression of FANCI, IFIH1 and low expression of PACRG was associated with shorter overall survival.” in the result part.

**Table 2:** Validation of the hub genes in TCGA database.

Hub gene	Methylation status	P value	Expression status	P value
**Hypomethylation/high-expression**				
FANCI	Hypomethylation	4.527e-4	High expression	6.049e-30
POSTN	Hypomethylation	4.268e-4	High expression	1.408e-20
IFIH1	Hypomethylation	9.634e-5	High expression	2.971e-8
**Hypermethylation/low-expression**				
ZMYND10	Hypermethylation	2.998e-6	Low expression	2.763e-5
PACRG	Hypermethylation	5.885e-4	Low expression	2.284e-15
POU2AF1	Hypermethylation	4.981e-8	Low expression	0.119

## Discussion

Exploring the potential mechanisms of NPC initiation and development will greatly benefit the diagnosis, treatment and prognosis assessment. With the development of sequencing technologies and microarray, we can easily screen the expression and methylation levels of thousands of genes simultaneously in the human genome. In this study, we identified 181 hypomethylation-high expression genes and 210 hypermethylation-low expression genes by multiple bioinformatics tools. Enrichment of these genes demonstrated that aberrant methylation indeed affects certain pathways and hub genes. Our findings can provide novel insight into the explanation of NPC pathogenesis.

The GO enrichment analysis demonstrated that the hypermethylated-low expression genes were involved mainly in controlling axoneme assembly, microtubule bundle formation, axonemal dynein complex assembly, cilium movement and cilium organization. However, the hypomethylated-highly expressed genes were involved in the regulation of endodermal cell differentiation, mitotic nuclear division, mitotic cell cycle process, chromosome segregation and cell cycle phase transition.

It has been described with several studies that alteration of gene expression in particular genes may affect the NPC tumor behavior, including proliferation capacity, apoptosis of affected cells, and cellular invasion and metastasis [21,22,23]. This result is consistent with association of invasion of tumoral cell and metastasis with abnormal cell adhesion and endodermal cell proliferation and division of nucleus [24]. Moreover, proliferation and apoptosis of tumoral cells are closely associated with defect in the mitotic cell cycle [25]. As for molecular function of GO analysis, hypomethylation/high-expression genes were enriched mainly in extracellular matrix structural constituent, receptor binding, cell adhesion molecule binding, tubulin binding and microtubule binding, while hypermethylation/low-expression genes were mostly enriched in microtubule motor activity, cytoskeletal protein binding, motor activity, chaperone binding and cytokine activity. Extracellular matrix (ECM), serves as a central element of the cancer cell niche, providing mechanical support and mediating cell-microenvironment interactions [24]. Significantly, collagens are one of the major proteins found within the ECM and have themselves been implicated in many aspects of neoplastic transformation. Therefore, it is consistent with the findings that active functions of these cellular processes through ECM were the main cause for tumor development, progression and metastasis. Tubulin binding, microtubule binding and microtubule motor activity were associated with decreased muscle function-mediated cytoskeleton remodeling in cancer development and progression [26,27,28].

Furthermore, the KEGG pathway of hypomethylated-highly expressed genes mainly involved in ECM receptor interaction, PI3K-Akt cell signaling pathway, cell-cell adhesion, protein digestion and absorption and Amoebiasis. Therefore activation in these signaling pathways plays an essential role in cell migration, differentiation and proliferation. As a contrast, hypermethylation-low expression genes were related to coagulation and complement cascades, hematopoiesis, leukocyte function (including, Leukocyte trans-endothelial migration, phagocytosis, and B-cell receptor (BCR) signaling pathway). BCR pathway is an important signaling pathway for B cell survival. Overactivation of several key kinases in BCR signaling pathway is involved in the B cell lymphoma (tumor development, and drug resistance).

The PPI network of hypomethylation-high expression genes was performed to provide an approach to the functional associations among them. The following core genes were selected: FANCI, POSTN and IFIH1. Fanconi anemia group I protein (FANCI) which plays an important role in repair of double strand DNA breaks through homologous recombination. FANCI also is very important for the repair of interstrand DNA cross-links (ICLs) and participating in recruitment to DNA repair sites [29]. Normal function of FANCI is required for maintenance of chromosomal stability. It regulates the S/G2 cell cycle checkpoint, following DNA damage [30]. A disorder affecting all bone marrow elements that may result to anemia, leukopenia and thrombopenia could be caused by FANCI mutation. Meantime, FANCI mutation is also related to malformations in heart, kidney and limbs, predisposition to cancer development and pigmentary changes in the skin. At the cell level, FANCI mutation is associated with susceptibility to DNA-damaging, instability in chromosome, and defect in DNA repair. FANCI is a negative regulator of Akt, thus it connects PI3K-Akt, and FA pathways which have oncogenic and tumor suppressing properties, respectively. FANCI mutation also results in chemotherapy drug resistance [29]. Our findings further support the potential of using FACNI as prognostic marker.

Periostin (POSTN) is an extracellular matrix protein secreted mainly by stromal cells and involved in the remodelling process of the tissue microenvironment, and neoplastic cell metastasis. In humans, POSTN is encoded as a ligand that interacts with alpha-V/beta-3 and alpha-V/beta-5 integrins to allow epithelial cells adhesion and migration. It is highly expressed in periosteum, perichondrium, periodontal ligament, muscular fascia, articular surface of cartilage and articular ligament, thus it is highly related to bone development, tissue repair, cardiovascular system and respiratory system. In addition, POSTN is also expressed in inflammation and various cancers. That’s why it promotes the metastasis of various cancers such as colon cancer and breast cancer [31]. Interferon-induced helicase C domain-containing protein 1(IFIH1), Innate immune receptor, serve as a cytoplasmic sensor for viral nucleic acids. IFIH1 involved in the antiviral cascades’ activation in response to viral infection (including encephalomyocarditis virus (EMCV) and Mengo encephalomyocarditis virus (ENMG), dengue virus (DENV), west Nile virus (WNV), and reovirus), via induction of Type-1 INF and proinflammatory cytokines. In addition, IFIH1 is involved in enhancing natural killer cell function and may play a role in tumor cell apoptosis. Therefore, FANCI, POSTN and IFIH1 may all be abnormally methylated genes that regulate the cell cycle and modulate cell proliferation in NPC.

About the hypermethylated/low-expression genes, ZMYND10, PACRG and POU2AF1 are the most prominent hub genes. Zinc finger MYND domain-containing protein 10 (ZMYND10), also known as BLU, is significantly down-regulated in several primary tumours such as neuroblastoma, esophageal squamous cell carcinoma and also malignancies in lung, breast, kidney. Some studies have found that ZMYND10 can promote cell apoptosis through PI3-K/PKB or Bcl2 family-mediated cell proliferation pathway. Over expression of ZMYND10 can reduce the migration, invasion and angiogenesis of nasopharyngeal carcinoma cells. ZMYND10 can also reduce the expression of CyclinD1, leading to cell cycle arrest. Parkin coregulated gene (PACRG) is co-expressed with Parkinson's disease-related Parkingene and shares a bidirectional promoter. It is closely related to the function of tubulin. PACRG regulates the movement of flagella and cilia, participates in sperm development, weak the toxicity of Parkin substrate and increase the survival rate of cells. PACRG is associated with several cancers, such as renal clear cell carcinoma, leukemia, glioma and so on. The abnormal expression of PACRG gene, promoter methylation may be related to tumorigenesis. POU Class 2 Homeobox Associating Factor 1 (POU2AF1) functions in the pre-B1-to-pre-B2 cell transition, and it is essential for pre-B-cell receptor (pre-BCR) and BCR signaling at multiple stages of B-cell development through its non-transcriptional regulation of SYK, a tyrosine kinase. Evidence indicates that POU2AF1 is associated with various types of lymphoid malignancies, colorectal cancer, Lynch syndrome, and survival in breast cancer.

These three genes are related to tumorigenesis, metastasis, and NPC tumor prognosis. In addition, survival analysis of hub genes demonstrated that while IFIH1, FANCI, and POSTN play a pro-oncogenic role, ZMYND10, PACRG and POU2AF1 genes were related to favorable survival in NPC.

## Conclusion

Our study combined the analysis of gene expression profiling microarrays and gene methylation profiling microarrays by bioinformatics analysis of available microarray data. This possibly eventuates to more reliable and precise screening results. Moreover, Our study provides a comprehensive bioinformatics analysis of aberrantly methylated DEGs that may be involved in the NPC tumorigenesis. We identified 6 mostly modified hub genes, including FANCI, POSTN, IFIH1, ZMYND10, PACRG and POU2AF1. These novel findings may provide insights for unravelling of the pathogenesis of NPC, and these potential genes may be used as optimal biomarkers to precisely detect and treat NPC. However, in our study, we only validated methylation in genes that were differentially expressed using the Oncomine and TCGA database. This necessitates further experiments to confirm our results in tumorigenesis in patient with NPC.

## Supporting information

S1 Table GSE53819expression matrix. (TXT)

## Abbreviations

BCR: B cell receptor
DEGs: Differentially expressed genes
DENV: dengue virus
DMGs: differential methylated genes
DMGs: Differentially methylated genes
dsDNA: Double-stranded DNA
EBV: Epstein-Barr virus
ECM: Extracellular matrix
EMCV: Encephalomyocarditis virus
ENMG: Encephalomyocarditis virus
FANCI: Fanconi anemia group I protein
ICLs: Interstrand DNA cross-links
IFIH1: Interferon-induced helicase C domain-containing protein 1
NPC: Nasopharyngeal carcinoma
PACRG: Parkin coregulated gene
POU2AF1: Class 2 Homeobox Associating Factor 1
PPI: Protein-protein interaction
RNase L: ribonuclease L
ssDNA: Single-stranded DNA
TCGA: The Cancer Genome Atlas
ZMYND10: Zinc finger MYND domain-containing protein 10
